# A multi-centre, double-blind, 12-week, randomized, placebo-controlled trial to assess the efficacy of adjunctive N-Acetylcysteine for treatment-resistant PTSD: a study protocol

**DOI:** 10.1186/s12888-020-02793-9

**Published:** 2020-08-06

**Authors:** Alice Maier, Anita Dharan, Gina Oliver, Michael Berk, Suzy Redston, Sudie E. Back, Peter Kalivas, Chee Ng, Richard A. Kanaan

**Affiliations:** 1 Department of Psychiatry, Austin Health, University of Melbourne, LTB10, 145 Studley Road, Heidelberg, VIC 3084 Australia; 2 Department of Psychiatry, The Melbourne Clinic, University of Melbourne, Richmond, VIC Australia; 3 IMPACT – the Institute for Mental and Physical Health and Clinical Translation, School of Medicine, Deakin University, Barwon Health, Geelong, Australia; 4 Orygen, The National Centre of Excellence in Youth Mental Health, Centre for Youth Mental Health, Florey Institute for Neuroscience and Mental Health and the Department of Psychiatry, The University of Melbourne, Melbourne, Australia; 5 Austin Health, Heidelberg, VIC Australia; 6 Department of Psychiatry and Behavioral Sciences, Medical University of South Carolina; Ralph H. Johnson VA Medical Center, Charleston, SC USA; 7 Department of Neuroscience, Medical University of South Carolina, Charleston, USA; 8 Ralph H Johnson VA Medical Center, Charleston, SC USA

**Keywords:** Clinical trial protocol, N-acetyl cysteine, Post-traumatic stress disorder, N-acetyl cysteine, Adjunctive therapy, Randomised clinical trial, Oxidative stress, Biomarker

## Abstract

**Background:**

Most patients with Posttraumatic Stress Disorder (PTSD) suffer residual symptoms following first-line treatment. Oxidative stress has been implicated in the pathophysiology of PTSD. N-acetylcysteine (NAC) is a precursor of the brain’s primary antioxidant, glutathione, and may diminish oxidative cellular damage. An 8-week pilot study of NAC in veterans with PTSD found that symptoms were significantly reduced in the NAC group compared to placebo. This study aims to confirm these findings with a larger sample in a double-blind, placebo-controlled trial to further explore the efficacy of NAC as an adjunctive therapy in treatment-resistant PTSD.

**Methods:**

A multicentre, randomised, double-blind, placebo-controlled trial for adult patients who still meet criteria for PTSD following first-line treatment. The intervention comprises either NAC as a fixed dose regime of 2.7 g/day (900 mg three times daily) administered orally for 12 weeks, or placebo. Standard care for PTSD will continue in addition, including other pharmacotherapies. Detailed clinical data will be collected at randomisation and weeks 4, 8, 12, 16, and 64 post-randomisation, with self-report measures completed weekly from baseline to 16 weeks and at 64 weeks post-randomisation. Blood-based biomarkers will be collected at baseline and 12 weeks to assess the mechanism of effect. The primary outcome measure will be change in Clinician-Administered PTSD Scale for DSM-5 at 12 weeks compared with baseline. Secondary outcomes will be change in quality of life, depression, anxiety, substance use and craving, and somatic symptoms. With 126 completed participants (63 per arm), the study is powered at 80% to detect a true difference in the primary outcome measure using a two-tailed analysis with alpha = 0.05, beta = 0.2.

**Discussion:**

This is the first multicentre, double blind, randomised, placebo-controlled trial of adjunctive NAC for treatment-resistant PTSD. NAC has an established safety profile, is readily available and easy to administer, and has a favourable tolerability profile, therefore making it an attractive adjunctive therapy. Inclusion of blood analyses to assess potential target engagement biomarkers of oxidative stress and neuroinflammation may help gauge the biological mechanisms of effect of NAC.

**Trial registration:**

ACTRN12618001784202, retrospectively registered 31/10/2018, URL: http://www.anzctr.org.au/Trial/Registration/TrialReview.aspx?id=376004.

## Background

Posttraumatic Stress Disorder (PTSD) is a condition characterized by re-experiencing of trauma, deterioration in cognition and mood, avoidance of triggers and hyper-arousal symptoms after experiencing severely threatening, traumatic events [1]. Large community surveys suggest that most people will experience at least one potentially traumatic event in their lives [2], however in a proportion of individuals, symptoms of psychological distress persist and progress to the development of PTSD. PTSD affects approximately 6% of the population, though rates are considerably higher for certain occupational groups such as emergency service workers and military personnel [3]. It is difficult to treat, with only a quarter of patients achieving full remission with first-line therapy [4].

PTSD is associated with substantial disability as symptoms are usually chronic [5] and accompanied by extensive functional decline, including high rates of unemployment [6], family and relationship difficulties [7], suicidality [8] and reduced quality of life [9]. PTSD is also frequently associated with a variety of comorbidities such as depression, substance abuse and psychosomatic disorders [1]. Recommended first-line treatments of PTSD in Australia are psychological, including trauma-focused psychotherapy, but they suffer from limited tolerability, and many patients discontinue treatment prematurely [10]. Selective-serotonin reuptake inhibitors (SSRIs) are the preferred pharmacological intervention for PTSD, either as alone or in conjunction with psychological therapies [11, 12]. SSRIs have demonstrated only moderate efficacy, however, with the majority of patients failing to achieve remission even when combined with psychotherapy [13, 14]. Those who do not respond to first-line treatment are usually considered treatment-resistant, and by this measure up to 70% of patients with PTSD fall into that category [15].

Several lines of evidence suggest that increased oxidative stress and neuroinflammation are implicated in the pathophysiology of PTSD [16–18]. Oxidative stress occurs when there are excess levels of free radicals, or inadequate antioxidants, potentially leading to cellular damage. Multiple studies in PTSD samples have demonstrated elevated levels of biomarkers of oxidative-stress, including anti-oxidant blood-based enzyme concentrations and altered gene expression [19, 20]. The brain’s primary antioxidant, glutathione, is dysregulated in patients with PTSD, [21] and downregulation of the Glutathione-S-Transferase Mu1 & 2 genes, responsible for encoding enzymes involved in conjugation reactions with glutathione, is considered the most reliable blood-based biomarker for PTSD [22]. Replenishing glutathione could be expected to diminish oxidative cellular damage, and hence potentially ameliorate PTSD symptoms and prevent long-term illness sequelae. However, glutathione is not orally bioavailable. In order to restore glutathione supply, its synthesis must be enhanced, for example by a precursor such as N-acetylcysteine (NAC).

Research indicates that NAC, or more likely its metabolites, can cross the blood-brain barrier [23, 24] and systemic delivery leads to a rise in brain glutathione [25]. While emerging evidence supports the use of NAC in the treatment of other psychiatric disorders [26, 27], this has yet to be thoroughly explored in PTSD. A small pilot study assessing NAC as an adjunct to cognitive-behavioural therapy in a group of US veterans with co-morbid substance use disorders demonstrated significant reductions in self-reported PTSD symptoms, co-morbid depressive symptoms and cravings for substance use compared to the placebo group. In view of these findings and the growing understanding of theoretical foundations of oxidation biology, NAC appears well placed as a potential adjunctive treatment for PTSD. It is well-tolerated [28, 29], low cost and is available without prescription. To date, NAC has yet to be evaluated in a large scale, randomised clinical trial as an adjuvant treatment for PTSD.

### Study objectives

This study aims to investigate the efficacy of adjunctive NAC for patients with treatment-resistant PTSD. It is hypothesised that NAC plus treatment as usual, will be superior to placebo plus treatment as usual in reducing clinician-assessed PTSD symptoms in an adult PTSD sample. It is also hypothesised that adjunctive NAC will result in a reduction in self-reported PTSD, depressive and somatic symptoms, substance use and cravings, and improvement in quality of life. This study will also explore blood-based biomarkers in PTSD to elucidate the mechanisms of action of NAC on PTSD symptoms.

## Methods

### Study design and setting

This study is a multi-site, 12-week, double-blind, randomised, placebo-controlled trial. The trial will be conducted at two tertiary mental-health centres in Australia which provide specialist PTSD treatment services: Austin Health’s public Psychological Trauma Recovery Service (PTRS) and The Melbourne Clinic (TMC) which is a private psychiatric hospital. Both sites acquired ethics approval to allow recruitment of participants from their clinical services and from the general community.

### Patient and public involvement

Patients or the public were not involved in the design, or conduct, or reporting, or dissemination plans of this study.

### Patients

Eligibility criteria are outlined in Table 1. The target population includes individuals aged 18 and older who meet Diagnostic & Statistical Manual of Mental Disorders - Fifth edition (DSM-5) diagnostic criteria for current PTSD despite having undergone a recommended course of treatment. A treatment course is defined as a full course of trauma-focused psychotherapy or a 6-week (minimum) course of pharmacotherapy (SSRI or SNRI). Participants may continue their usual treatments during the study. Participants must have the capacity to consent to the study and to follow its instructions and procedures.

**Table 1:** Inclusion and Exclusion Criteria

Inclusion criteria	Exclusion criteria
Age ≥ 18 and able to provide written informed consent	Already taking NAC, selenium or vitamin E History of anaphylactic reaction to NAC
**Current PTSD** Meets criteria for DSM-5 diagnosis of PTSD according to CAPS-5 monthly version	Known or suspected clinically unstable systemic medical disorder History of epilepsy Current asthma Recent gastrointestinal ulcers
Treatment resistant PTSD defined as (a) Minimum 1x trial of an antidepressant (SSRI or SNRI) for minimum 6 weeks without adequate response OR (b) completed a course of trauma-focused psychotherapy	Currently pregnant or breastfeeding Planning to conceive a child during the trial or 3 months following
If currently taking an antidepressant medication • Minimum of 6-weeks if recently initiated • Dose must be stable for minimum 2 weeks	History of psychotic illness

*NAC* N-acetyl cysteine, *PTSD* Posttraumatic Stress Disorder, *DSM-5* Diagnostic & Statistical Manual of Mental Disorders Fifth edition, *CAPS-5* Clinician-Administered PTSD Scale for DSM-5, *SSRI* Selective-serotonin reuptake inhibitor, *SNRI* Serotonin and norepinephrine reuptake inhibitor

### Sample size calculation and recruitment target

The target sample size is 126 participants (63 per arm). For a two-tailed analysis with alpha = 0.05, beta = 0.2, the study is powered at 80% to detect a true difference in CAPS-5 scores between the NAC and placebo groups of 10+/− 20 points. In our pilot trial, effect sizes for PTSD symptoms as well as all secondary measures exceeded this threshold. Using a conservative attrition rate of one-third, we will need to recruit 190 participants to have 126 complete the trial and follow-up.

**Fig. 1:**
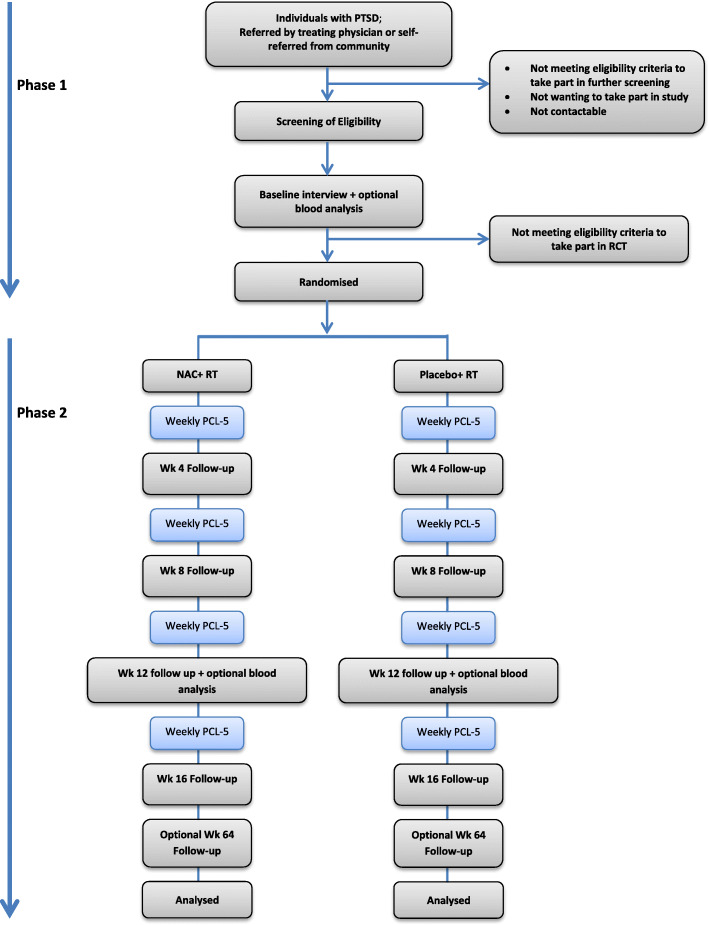
Trial Flow Diagram. NAC – N-Acetyl Cysteine; RT – Routine treatment; PCL-5 – PTSD Checklist 5

The study aims to recruit participants referred by their treating clinician or self-referred from the community. A summary of the participant flow through the study is shown in Fig. 1.

### Screening phase and baseline assessment

Individuals with PTSD who express interest in participating in the study will be contacted by a trial clinician and screened according to the eligibility criteria outlined in Table 1 via a brief telephone interview.

After initial telephone screening, eligible participants will be invited to a face-to-face or videoconference 90-min clinical interview. After obtaining IRB-approved written informed consent, a diagnosis of PTSD will be confirmed through the Clinician-Administered PTSD Scale for DSM-5 (CAPS-5) – monthly version, which is the gold-standard in PTSD assessment. Participants will be excluded if they do not meet diagnostic criteria for current PTSD. If patients are deemed eligible and are enrolled in the study, this interview will constitute part of the baseline assessment.

The baseline assessment will also include data collection via clinician-administered and self-reported questionnaires regarding psychiatric comorbidities, PTSD symptoms, quality of life, substance use and somatic symptoms (see Table 2). Detailed clinical and demographic information will be collected on all participants at the baseline clinical interview. This will include age, gender, marital status, years of formal education, service history, receipt of state benefits, ethnicity, and country of birth.

**Table 2:** Primary and secondary outcomes according to time-point

Outcome	Measure	Trial							Post-trial		
		Baseline	W 1–3	W 4	W 5–7	W 8	W 9–11	W 12	W 13–15	W 16	W 64
**Primary Outcome**											
*Trauma history, PTSD diagnosis and symptom severity*	**CAPS-5** (monthly version; 30-item observer rated questionnaire, corresponding to the *DSM-5* diagnosis for PTSD) [30]	X						X		X	X
*PTSD symptom severity*	**CAPS-5** (weekly version)	X		X		X		X		X	
**Secondary Outcomes**											
*Depression & Anxiety*	**HADS** (14 item self-report screening measure of anxiety and depression, designed for use in medical settings) [31]	X		X		X		X		X	X
	**HAM-D** (17 item observer rated scale; evaluates core symptoms of depression) [32]										
*Self-rated PTSD symptoms*	**PCL-5** (20 item self-report measure of PTSD symptoms) [33]	X	X	X	X	X	X	X	X	X	X
*Somatic symptoms*	**PHQ-15** (15 item self-report measure of degree to which one has been bothered by somatic symptoms over the past month) [34]	X		X		X		X		X	X
*Quality of life*	**WHO-QOL BREV** (26 item measure of subjective quality of life) [35]	X		X		X		X		X	X
*Alcohol use*	**AUDIT** (Participant answer ten-items relating to frequency of symptoms and behaviours associated with alcohol misuse) [36]	X		X		X		X		X	X
*Substance craving*	**Visual Analogue Scale** (to assess substance craving over the past week. Participants will rate 5 items using anchors of 0 (“not at all”) to 10 (“extreme” or “all the time”)	X		X		X		X		X	X
*Biomarkers of oxidative stress*	**Optional blood analysis** (IL-6, TNF-alpha, TBARS, MDA, BDNF)	X						X			
**Other Measures**											
*Other psychiatric comorbidities*	**MINI V 5.0** (Semi-structured interview to assess habitual substance use and psychiatric comorbidities, including psychotic disorders) [37]	X									
*Demographic Information*	Questionnaire	X									
*Adverse effects*	Self-report			X		X		X			

Indicates clinical interview

Participants will be provided with a signed copy of their information and consent form which includes written information regarding study protocol, purpose, data handling, participant confidentiality, potential side-effects and their right to withdraw from the study at any time.

### Allocation

Randomisation will take place only once participants have undertaken the baseline assessment and are confirmed eligible for the trial. If all inclusion and exclusion criteria are satisfied, trial participants will be randomised at a 1:1 ratio to the NAC (treatment) or placebo arm. Random block allocation of medication packs will be in a four-to-a-block design, randomly generated by a computer program. Randomisation will be carried out by the clinical trial pharmacy service at the trial site. Allocation will be concealed from participants and all other research staff as the study is double blinded.

A fixed dose regime of 2.7 g/day of NAC will be administered as 900 mg three times daily for 12 weeks. Previous pilot studies have demonstrated this dose is efficacious and well-tolerated [28, 29]. The NAC and placebo are either supplied as effervescent tablets or as capsules. The trial medications will be supplied on a monthly basis (to coincide with clinical interviews) and participants will be instructed to return all packaging to allow capsule counts by the trial monitor and the trial pharmacist. Adherence will be assessed by pill counts of returned medication packs. Participants will also be required to complete a daily pill diary to document treatment compliance. The trial medications will be supplied, and returned containers counted, by the trial pharmacist.

### Blinding

To facilitate the double-blinding process, the trial medications (both NAC and placebo) will be dispensed in identical numbers and tablet forms in sealed containers, and the placebo containers will be specially treated with microgram levels of NAC dust to produce its characteristic smell. Placebo will be manufactured according to European Medicine Agency (EMA) guidelines. We will require research workers to indicate if they have become unblinded so that outcome assessments can be undertaken by blinded assessors in addition to any established treatments for their PTSD.

### Follow-up assessments

Following baseline assessment and enrolment in the trial, participants will be assessed with clinical interviews at weeks 4, 8 and 12 of the trial. Post-trial interviews are conducted at 16 weeks and 64 weeks to assess retention of any treatment effects. At each follow-up assessment, clinician-rated PTSD symptoms, and self-reported PTSD symptoms as well as psychiatric comorbidities, quality of life, substance use and somatic symptoms are assessed (see Table 2). In between follow-up interviews (from Baseline to Week 16), participants will complete weekly self-report questionnaires regarding their PTSD symptoms. Follow-up interviews will take about 60 min to complete; the weekly self-report questionnaire take approximately 5 min.

### Outcomes

Our primary evaluation of treatment efficacy is the reduction of observer-rated PTSD symptom severity, operationalised as a reduction CAPS-5 severity scores. Secondary objectives include evaluation of NAC to reduce self-reported symptoms of (1) PTSD, (2) depression and anxiety, (3) substance use, (4) craving and (5) somatic symptoms. Evaluation of treatment efficacy will be assessed over the entire trial period. Retention of treatment effects will be assessed at weeks 16 and 64 post-randomisation. The safety and tolerability of the intervention will be evaluated by self-report.

Participants may elect to provide a blood sample at baseline and Week 12 assessments. Blood based biomarkers of oxidative stress will be evaluated as exploratory outcomes. Blood analysis will include measurement of IL-6 (interleukin-6), TNF-alpha (tumour necrosis factor alpha), TBARS (thiobarbituric acid reactive substances), MDA (malondialdehyde) and BDNF (brain-derived neurotrophic factor). Modulation of these blood-based proteins will be correlated with treatment response. Participants will not be fasting prior to the blood collection. BD vacutainer SST II advance tubes (5 mL) will be used to collect the sample and then allowed to rest for 30 min prior to being centrifuged at 1300 g for 15 mins. The supernatant (serum) will be extracted and aliquoted into 2 × 1.8 ml tubes. All samples will be stored at − 80 degrees Celsius until tested.

### Participant safety and withdrawal

Adverse effects will be assessed each week and reviewed by the study team as well as the Data Safety Monitoring Board, comprising the investigators. Though serious adverse events with NAC are extremely rare, patients will be advised to seek appropriate emergency medical help in such an instance. For less serious events, participants will be advised to contact their treating physician as usual. Participants will be withdrawn from the trial under the following conditions: (i) failure to take the trial medication for seven consecutive days; (ii) cessation of effective contraception or pregnancy; and (iii) withdrawal of consent or emergence of adverse events (as determined by the medical investigators or decision of the participant). All data will be retained in the intention-to-treat analysis.

### Standardised medical care

Standard care for participants will involve their regular, medical appointments at variable intervals. The additional procedures involve meeting with the research clinician in outpatients of the clinical services – where possible to coincide with scheduled appointments – at 4-weekly intervals, where they will engage in a clinical interview, and complete self-report questionnaire items. Participants will also be asked to complete one self-report questionnaire, each week on their own.

### Interrater reliability

All research staff undertaking clinical assessments will be trained in the assessments prior to commending recruitment. All clinical research staff will undertake observed interviews on a yearly basis to ensure compliance with standardised administration guidelines and complete reliability and inter-rater reliability assessments.

### Statistical analysis

All analyses will be conducted by our independent trial statistician and will include all randomised participants with at least one post-baseline observation (modified intention to treat). The statistician will be blind to group allocation (triple blind design). Differences between study arms at baseline will be assessed using either chi-squared or Fisher’s exact test for categorical data and either student t–test or Mann-Whitney U test for continuous data. Non-parametric statistics will be used when assumptions for parametric methods are violated. Effect sizes will be calculated using Cohen’s guidelines. The primary efficacy analysis will assess the impact of the treatment on group differences for the primary outcome measure (CAPS-5) over the entire study period and use a likelihood based mixed-effects model, repeated measures approach (MMRM). The MMRM model includes the categorical effects of treatment, investigator, visit, and treatment-by-visit interaction, as well as the continuous, fixed covariates of baseline score and baseline score-by-visit interaction.

### Data handling and monitoring

Paper copies of the questionnaires will be kept in a locked filing cabinet in the Department of Psychiatry of the clinical services, in a room kept locked. The data will be entered into a computer in re-identifiable form, with the code kept in a separate file. Computer files will be kept on a password-protected computer in the department of psychiatry in a room kept locked at night. All files will be kept for 15 years before secure disposal.

All blood samples will be securely stored at the Austin Health Pathology Department in a monitored, trial specific freezer at − 80 degrees Celsius and labelled in a re-identifiable format before being transferred to the Flory Institute of Neuroscience and Mental Health for analysis. Following analysis, all blood will be securely destroyed by the Flory Institute of Neuroscience and Mental Health. Blood samples collected for the purposes of this study that are not transferred to the Flory Institute of Neuroscience and Mental Health for analysis will be destroyed after a period of 7 years.

## Discussion

This study described the design and methodology of the first multi-centre, randomised, double-blind, placebo-controlled 12-week trial evaluating the efficacy of NAC as an adjuvant therapy in treatment-resistant PTSD. Despite recent advancements in evidence-based interventions for PTSD, many patients continue to suffer substantial residual symptoms post-treatment [13, 14, 38]. Accumulating evidence supports the potential efficacy of NAC in symptom reduction and functional improvements across a range of psychiatric and neurological disorders [26, 27, 39]. We hypothesise that when delivered as an adjuvant therapy, NAC will reduce PTSD and related symptomatology greater than treatment as usual.

Oral NAC is widely available over the counter as an inexpensive nutritional supplement and has demonstrated low rates of adverse effects [26, 39, 40]. These factors support the use of NAC in clinical settings as a safe, inexpensive, easily administered and non-invasive supplementary treatment strategy for PTSD. However, recommendations regarding the use of NAC in psychiatric disorders are limited by the small number, small sample sizes and quality of existing studies [26]. As such, large controlled trials assessing the efficacy of NAC in specific disorders are essential to establish its efficacy. The anticipated sample size of the present study is likely to exceed many previous clinical studies [41], thus increasing the statistical power and generalizability of the study findings. The large sample size, and randomised placebo-controlled design are significant methodological strengths in the current study. A further strength is the assessment of clinical outcomes at 4- and 52-weeks post-trial completion. Previous clinical trials have produced mixed results regarding the retention of treatment benefits of NAC in other psychiatric disorders, with some effects either retaining or emerging at 4 weeks post-trial completion and others being lost [29, 42].

While NAC has been implicated as an effective adjunctive treatment for many psychiatric conditions, the mechanism of effect has not yet been fully elucidated. The therapeutic effects of NAC are likely mediated via its ability to modulate numerous pathophysiological processes including oxidative stress, neurogenesis and apoptosis, mitochondrial dysfunction, neuroinflammation and dysregulation of glutamate and dopamine [26]. Determining the underlying mechanisms of NAC in PTSD is helpful, not only to understanding the underlying neurobiology of the illness but to discover additional therapies that may operate on similar pathophysiological pathways. Of key interest in the current study is NAC’s ability to replenish glutathione and thereby diminish oxidative stress and neuroinflammation, which are well established biomechanisms in PTSD and its other psychiatric comorbidities [16–18]. While studies have speculated on the mechanisms of action of NAC on psychiatric symptoms [26, 40] few have directly assessed blood-based biomarkers of oxidative stress and neuroinflammation in the context of treatment with NAC. The inclusion of blood-based biomarkers of oxidative stress and neuroinflammation is therefore a key methodological advantage of the present study.

A final advantage for this study is the heterogeneity of the sample. The broad inclusion and limited exclusion criteria mean that participants experiencing a range of psychiatric comorbidities will be eligible, and the adjuvant approach means that it will be available to most individuals with PTSD. To overcome the lack of specificity incurred by the more ecologically valid inclusion criteria, this study will thoroughly characterise psychiatric comorbidities and medical histories at baseline, so as to be able to distinguish whether treatment responses vary between presentations. Use of the gold standard CAPS-5 PTSD diagnostic tool will also enable the study to characterise trauma histories, and establish whether treatment responses differ between trauma types. This is particularly important, given that PTSD symptom severity and treatment response rates may differ between those with a history of prolonged developmental trauma and those with combat related PTSD [43–46].

## Data Availability

Anonymised, participant level data will be available from the corresponding author on reasonable request.

## Abbreviations

PTSD: Posttraumatic Stress Disorder
PTRS: Posttraumatic Recovery Service
TMC: The Melbourne Clinic
NAC: N-acetylcysteine
CAPS-5: Clinical Administered PTSD scale for DSM-5
